# Clinicopathological Correlation of Polyomavirus Nephropathy in Renal Allograft Recipients According to the Banff 2018 Classification

**DOI:** 10.7759/cureus.50910

**Published:** 2023-12-21

**Authors:** Tamkan Junyangdikul, Ngoentra Tantranont, Thanaporn Chaiyapak, Attapong Vongwiwatana, Boonyarit Cheunsuchon

**Affiliations:** 1 Department of Pathology, Faculty of Medicine Siriraj Hospital, Mahidol University, Bangkok, THA; 2 Division of Nephrology, Department of Pediatrics, Faculty of Medicine Siriraj Hospital, Mahidol University, Bangkok, THA; 3 Division of Nephrology, Department of Medicine, Faculty of Medicine Siriraj Hospital, Mahidol University, Bangkok, THA

**Keywords:** transplant infection, transplant pathology, renal pathology, histologic classification, polyomavirus nephropathy

## Abstract

Background: Polyomavirus nephropathy (PVN) is a rare kidney disease caused by the BK virus, a strain of polyomavirus. The disease primarily affects transplant recipients, which is related to intensive immunosuppression protocol and can lead to kidney allograft failure.

Objectives: The objective of this study is to analyze histopathological features of PVN using the Banff 2018 PVN classification and to determine clinical features and outcomes of patients with PVN in each histologic class.

Materials and methods: The study included 44 patients who had been diagnosed with PVN by renal allograft biopsy in a large tertiary care hospital in Thailand from January 2011 to January 2020. The kidney biopsy slides were reviewed for Banff 2018 PVN classification and other histologic features. Patient demographic information, clinical data, and laboratory results were retrospectively collected.

Results: Nine (20.45%), 27 (61.36%), and eight (18.18%) cases of PVN were Class I, Class II, and Class III, respectively. The time from transplant to PVN diagnosis for Classes I, II, and III was four, 19, and 33.5 months, respectively. Class III had the worst clinical outcomes in terms of deterioration of allograft function, the lowest rate of resolution, and the highest rate of graft failure.

Conclusions: PVN classification provides prognostic information in renal allograft biopsy. Our study confirmed the validity of the three-tier histologic PVN classification put forward by the Banff Working Group in 2018.

## Introduction

Polyomavirus nephropathy (PVN) is a disease arising from polyomavirus infection in kidneys and is most commonly found in renal transplant patients. The disease is caused by BK virus, a strain of polyomavirus, hence the other name for infection is BK virus nephropathy. Most adults had exposure to the virus in childhood, with nearly 90% seroprevalence [1]. PVN can affect 1-10% of renal transplant patients [2,3]. The disease is associated with intensive immunosuppression protocols used in organ transplant patients. PVN poses a significant risk factor for renal allograft loss [4].

Although polyomavirus can be detected in blood and urine, renal allograft biopsy is the gold standard for the diagnosis of PVN [5]. The Banff Group on Allograft Pathology introduced a histologic classification of PVN in 2018 to standardize histopathologic diagnosis and facilitate communication among transplant nephrologists and renal pathologists [6]. This novel three-tier histologic classification was designed to reflect three clinical parameters: (1) clinical presentation at the time of PVN diagnosis, (2) allograft function during follow-up, and (3) allograft loss [6]. Banff 2019 criteria for renal allograft pathology added the PVN classification as category 5 [7]. The classification has been proven to be useful in several cohorts [2,8]. Our study aims to validate the usefulness of Banff PVN histologic classification by clinicopathological correlation and outcome prediction in our patients.

## Materials and methods

The study received approval from the Institutional Review Board of the Faculty of Medicine Siriraj Hospital, Mahidol University (COA No. Si 058/2022). A total of 44 cases (97 biopsy specimens) of PVN were retrieved from the laboratory information system of the Department of Pathology, Faculty of Medicine Siriraj Hospital between January 2011 and January 2020.

Clinical data were collected and reviewed from the electronic medical records. The clinical data included age, gender, underlying disease, native kidney disease, donor source, warm ischemic time, cold ischemic time, anastomosis time, time from kidney transplant to PVN diagnosis at index biopsy, time from PVN diagnosis at index biopsy to graft failure, other diagnosis at index biopsy, and treatment protocols for PVN (immunosuppressant reduction, cidofovir, ciprofloxacin, and intravenous immunoglobulin). Serum creatinine levels were collected within three months before PVN diagnosis with median values defined as baseline levels, and subsequent median values at 0-12, 13-24, and after 24 months of follow-up.

PVN is categorized into three classes (Class I, Class II, and Class III) (Table 1) [6], using the pvl score (intrarenal polyomavirus load level) combined with the ci score (interstitial fibrosis), as shown in Table 1; pvl score 1: the presence of positive tubule up to 1% of all tubules; pvl score 2: the presence of positive tubule more than 1%-10% of all tubules; pvl score 3: the presence of positive tubule more than 10% of all tubules; “positive tubule” defined as the presence of intranuclear viral inclusion body or positive SV-40 immunostaining in at least one cell per tubular cross-section; ci score 0: interstitial fibrosis in up to 5% of the cortical area; ci score 1: interstitial fibrosis in 6%-25% of the cortical area (mild interstitial fibrosis); ci score 2: interstitial fibrosis in 26%-50% of the cortical area (moderate interstitial fibrosis); ci score 3: interstitial fibrosis in >50% of the cortical area (severe interstitial fibrosis).

**Table 1:** Polyomavirus nephropathy, Banff 2018 classification.

Class I	Class II	Class III
pvl 1 and ci 0-1	pvl 1 and ci 2-3	pvl 3 and ci 2-3
	pvl 2 and ci 0-3	
	pvl 3 and ci 0-1	

Histopathologic review was conducted by two renal pathologists (BC, NT). The histopathologic review included PVN diagnosis, classification (pvl score combined with ci score), and other diagnoses (graft rejection, acute tubular injury, glomerulonephritis, interstitial fibrosis, tubular atrophy, and glomerulosclerosis).

Statistical analysis was performed using percentage for categorical data, mean ± standard deviation for numerical data with normal distribution (age and time from PVN diagnosis at index biopsy to graft failure), and median (interquartile range: 1-3) for numerical data with non-normal distribution (all remaining numerical data). The comparison between percentage, mean, and median was conducted using the chi-square test, ANOVA, and Kruskal-Wallis test, respectively.

## Results

Incidence and pathology of polyomavirus nephropathy

**Figure 1:**
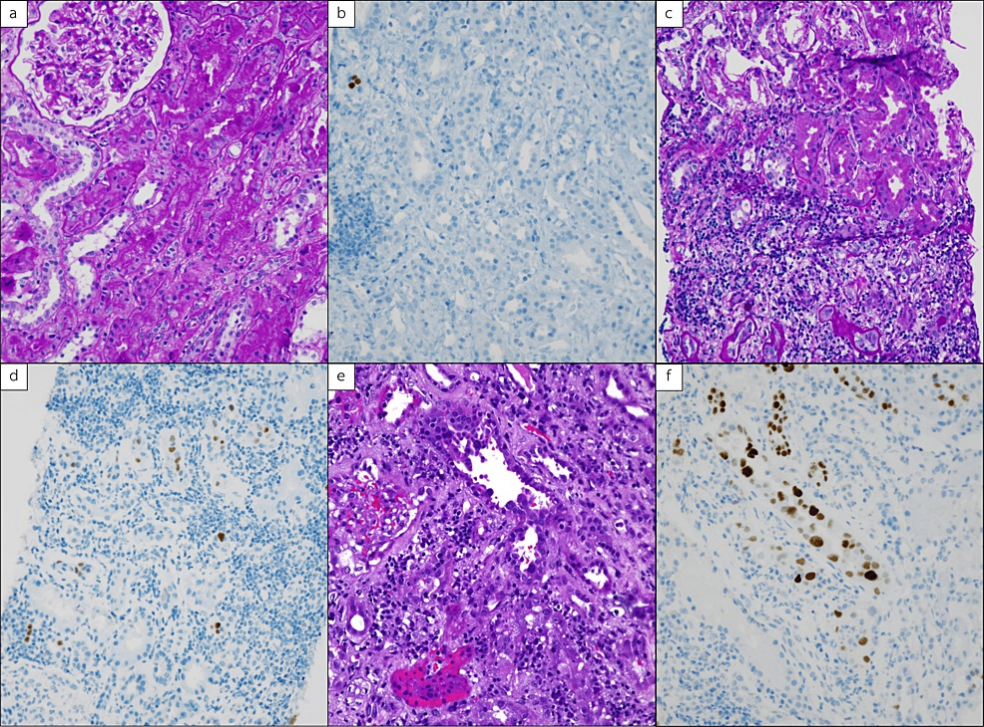
Histological findings in each Banff 2018 polyomavirus nephropathy classification. (a, c, e) Hematoxylin & eosin, x200. (b, d, f) SV40-T stain, x200. (a, b) Class I (pvl 1, ci 0). (c, d) Class II (pvl 2, ci 1). (e, f) Class III (pvl 3, ci 3).

**Figure 2:**
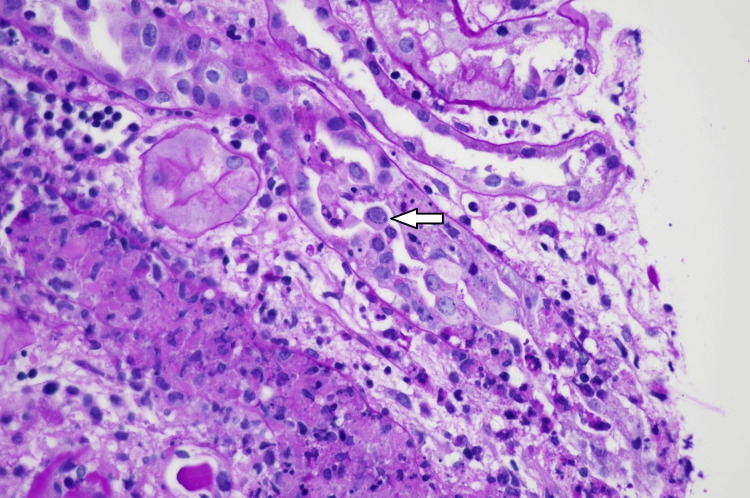
Tubular cell with intranuclear viral inclusion (arrow). Severe tubular injury and interstitial mononuclear cell infiltration were noted (hematoxylin & eosin, x400).

A total of 44 cases of PVN were diagnosed in our institute between January 2011 and January 2020. The incidence was 3.2% in biopsies for allograft dysfunction during the period of study. PVN cases increased in 2014 (four/year), peaked in 2015 (nine/year) and 2016 (nine/year), and declined thereafter. The cases were classified into three classes: Class I (nine cases, 20%), Class II (27 cases, 61%), and Class III (eight cases, 18%). Figure 1 shows examples of the pathology of PVN in each class. Figure 2 shows intranuclear viral inclusion with ground glass appearance. The definition is detailed in the materials and method section.

Patients’ demography and clinical characteristics

Baseline patient characteristics are shown in Table 2. Males were slightly more prominent (59%) but not statistically significant. The mean age was 41 years, with no statistical difference between the classes. The most common age group was 41-60 years old, with more than 50% of total cases. Hypertension was the most common co-morbidity (58%).

**Table 2:** Histologic classification and patients’ baseline characteristics. * Mean ± SD. CMV: cytomegalovirus.

Characteristics	N	Class I	Class II	Class III
Gender	N = 44	N = 9	N = 27	N = 8
Male (%)	26 (59.09)	8 (88.89)	14 (51.85)	4 (50)
Female (%)	18 (40.90)	1 (11.11)	13 (48.15)	4 (50)
Age (years)*	N = 44	N = 9	N = 27	N = 8
	41.48 ± 15.01	41.89 ± 13.67	39.22 ± 16.78	48.63 ± 6.95
≤20 (%)	6 (13.64)	1 (11.11)	5 (18.52)	0 (0)
21-40 (%)	10 (22.73)	1 (11.11)	8 (29.63)	1 (12.5)
41-60 (%)	24 (54.54)	7 (77.78)	10 (37.04)	6 (75)
61-80 (%)	4 (9.09)	0 (0)	3 (11.11)	1 (12.5)
Underlying disease	n = 38	n = 9	n = 23	n = 6
Diabetes mellitus, N (%)	6 (15.79)	1 (11.11)	3 (13.04)	2 (8.70)
Hypertension, N (%)	22 (57.89)	7 (77.78)	10 (43.48)	5 (83.33)
Dyslipidemia, N (%)	16 (42.11)	3 (33.33)	10 (43.48)	3 (13.04)
Obesity, N (%)	2 (5.26)	1 (11.11)	1 (4.35)	0 (0)
Coronary artery disease, N (%)	1 (2.63)	0 (0)	1 (4.35)	0 (0)
Valvular heart disease, N (%)	2 (5.26)	0 (0)	2 (8.70)	0 (0)
CMV infection, N (%)	10 (26.32)	6 (66.67)	3 (13.04)	1 (16.67)
Tuberculosis, N (%)	2 (5.26)	2 (22.22)	0 (0)	0 (0)
Chronic hepatitis B, N (%)	3 (7.89)	1 (11.11)	2 (8.70)	0 (0)
Chronic pancreatitis, N (%)	1 (2.63)	0 (0)	1 (4.35)	0 (0)
Hyperparathyroid, N (%)	5 (13.16)	3 (33.33)	2 (8.70)	0 (0)
Autoimmune diseases (rheumatoid arthritis, systemic lupus erythematosus), N (%)	3 (7.89)	0 (0)	1 (4.35)	2 (8.70)
Myelodysplastic syndromes, N (%)	1 (2.63)	0 (0)	1 (4.35)	0 (0)
Angioimmunoblastic T-cell lymphoma, N (%)	1 (2.63)	0 (0)	1 (4.35)	0 (0)
Major depressive disorder, N (%)	1 (2.63)	0 (0)	0 (0)	1 (4.35)
No underlying disease, N (%)	7 (18.42)	0 (0)	6 (26.09)	1 (4.35)

The most common causes of end-stage renal disease (ESRD) were IgA nephropathy (15%) and chronic glomerulonephritis (15%). IgA nephropathy was equally identified as the cause of ESRD in all classes, while chronic glomerulonephritis was only identified in Class II (six cases). Deceased donors were slightly more prominent (58%), without statistical significance. There were no living non-related donors in this study. Warm ischemic time, cold ischemic time, and anastomosis time were not statistically different between classes (Table 3).

**Table 3:** Histologic classification and transplantation parameters. * Median (IQR).

Parameters	N	Class I	Class II	Class III
Causes of end-stage renal disease	N = 39	N = 9	N = 23	N = 7
IgA nephropathy, N (%)	6 (15.38)	2 (22.22)	2 (8.70)	2 (28.57)
Chronic glomerulonephritis, N (%)	6 (15.38)	0 (0)	6 (26.09)	0 (0)
Polycystic kidney disease, N (%)	3 (7.69)	1 (11.11)	2 (8.70)	0 (0)
Obstructive nephropathy, N (%)	3 (7.69)	2 (22.22)	1 (4.35)	0 (0)
Lupus nephritis, N (%)	2 (5.13)	0 (0)	1 (4.35)	1 (14.29)
Diabetes nephropathy, N (%)	2 (5.13)	1 (11.11)	0 (0)	1 (14.29)
Hypertensive nephropathy, N (%)	1 (2.56)	0 (0)	1 (4.35)	0 (0)
Nephrotic syndrome, N (%)	1 (2.56)	0 (0)	1 (4.35)	0 (0)
Focal segmental glomerulosclerosis, N (%)	1 (2.56)	0 (0)	0 (0)	1 (14.29)
Alport syndrome, N (%)	1 (2.56)	1 (11.11)	0 (0)	0 (0)
Autosomal dominant hypocalcemia type 1, N (%)	1 (2.56)	0 (0)	1 (4.35)	0 (0)
Methylmalonic acidemia, N (%)	1 (2.56)	0 (0)	1 (4.35)	0 (0)
Unknown cause, N (%)	11 (28.21)	2 (22.22)	7 (30.43)	2 (28.57)
Donor type	N = 43	N = 9	N = 27	N = 7
Deceased donor, N (%)	25 (58.14)	8 (88.89)	12 (44.44)	5 (71.43)
Living related donor, N (%)	18 (41.86)	1 (11.11)	15 (55.56)	2 (28.57)
Kidney transplant operation				
Warm ischemic time (minute)*	22 (N = 11)	13.5 (N = 8)	5 (N = 1)	37.5 (N = 2)
Cold ischemic time (minute)*	827 (N = 25)	902 (N = 8)	600 (N = 13)	695.5 (N = 4)
Anastomosis time (minute), median*	44 (N = 27)	45.5 (N = 8)	45.5 (N = 16)	30 (N = 3)

Time to diagnosis and treatment

The time from transplant to PVN diagnosis of Class III was longer than that of Class II and Class I. On the other hand, the time from PVN diagnosis to graft failure was similar among the three classes (Table 4). For treatment protocol, immunosuppression reduction was performed in 79% of cases. Cidofovir was administered in 58% of cases. Class III cases (86%) were more likely to receive cidofovir than Class II and Class I cases (33% and 59%, respectively). Ciprofloxacin was administered in 89% of cases (100%, 91%, and 71% in Classes I, II, and III, respectively). Intravenous immunoglobulin was administered in 10% of cases, only in Class II and Class III.

**Table 4:** Time to diagnosis, graft failure, and treatment. * Mean ± SD. PVN: polyomavirus nephropathy.

	n	Class I	Class II	Class III
Time: Kidney transplant - PVN diagnosis (month)*	N = 44, 16 (5.75-30.5)	N = 9, 4 (3-10)	N = 27, 19 (7-29)	N = 8, 33.5 (15.5-48.75)
Time: PVN diagnosis - Graft failure (month)*	N = 13, 34.15 ± 23.86	N = 3, 47.33 ± 15.63	N = 6, 25.67 ± 20.14	N = 4, 37 ± 33.33
Concurrent diagnosis - Acute antibody-mediated rejection, N (%)	N = 44, 3 (6.82)	N = 9, 0 (0)	N = 27, 1 (3.70)	N = 8, 2 (25)
Acute T cell-mediated rejection, N (%)	1 (2.27)	0 (0)	1 (3.70)	0 (0)
Acute tubular injury, N (%)	1 (2.27)	1 (11.11)	0 (0)	0 (0)
Focal segmental glomerulosclerosis, N (%)	1 (2.27)	0 (0)	0 (0)	1 (12.5)
PVN treatment protocol				
Immunosuppression protocol	N = 34	N = 9	N = 20	N = 5
Reduction, N (%)	27 (79.41)	8 (88.89)	15 (75)	4 (80)
Unchanged, N (%)	2 (5.88)	0 (0)	1 (5)	1 (20)
Increase, N (%)	5 (14.70)	1 (11.11)	4 (20)	0 (0)
Antiviral therapy	N = 38	N = 9	N = 22	N = 7
Cidofovir, N (%)	22 (57.89)	3 (33.33)	13 (59.09)	6 (85.71)
Ciprofloxacin, N (%)	34 (89.47)	9 (100)	20 (90.90)	5 (71.43)
Intravenous immunoglobulin, N (%)	N = 38	N = 9	N = 23	N = 6
	4 (10.53)	0 (0)	3 (13.04)	1 (16.67)

**Figure 3:**
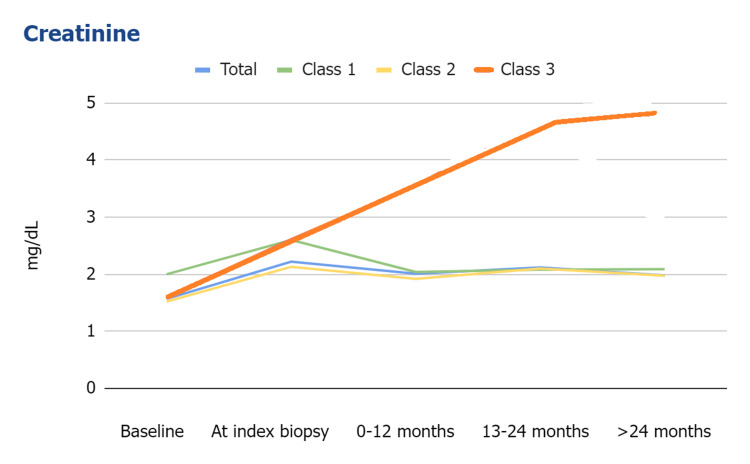
Follow-up allograft function in patients with polyomavirus nephropathy. * Median (IQR).

Although the serum creatinine at index biopsies was higher than baseline in all classes, 41% of patients presented with stable renal function at the time of PVN diagnosis. Class III showed a more rapid elevation of serum creatinine compared to Class I and Class II (Figure 3).

**Figure 4:**
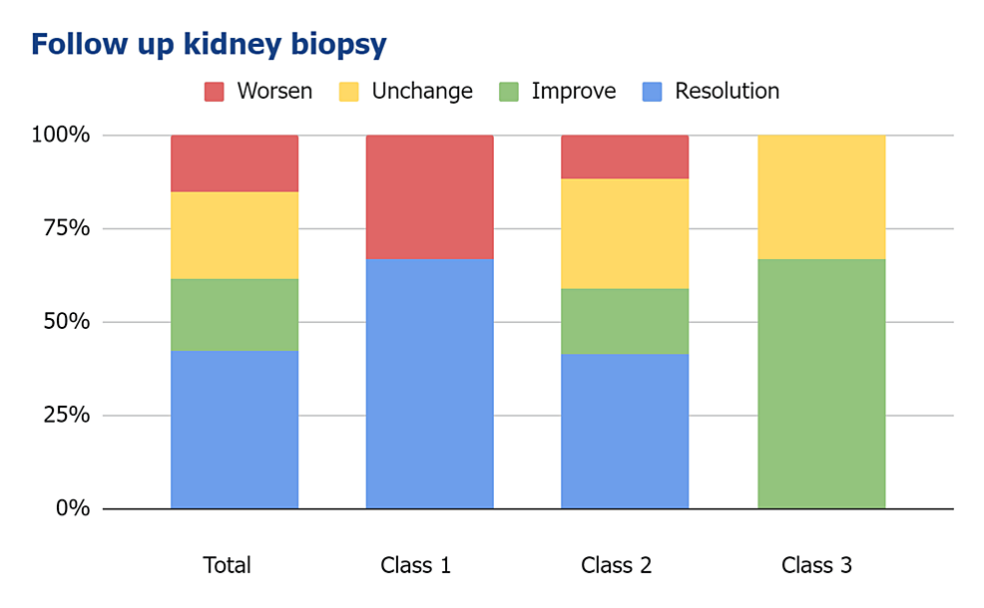
Polyomavirus nephropathy status of follow-up allograft biopsies.

**Figure 5:**
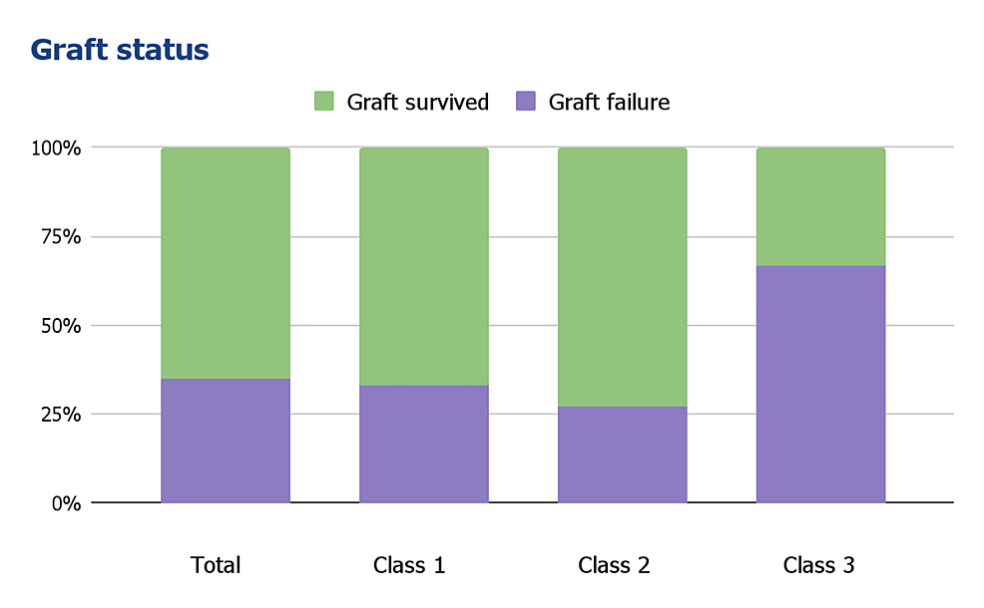
Allograft outcome.

The follow-up allograft biopsies were grouped into four PVN statuses: resolution (no PVN), improvement (improvement in PVN class), unchanged (unchanged PVN class), and worsened (progression of PVN class). Among the total cases, 42% showed resolutions, 19% showed improvements, 23% were unchanged, and 15% were worsened. The resolution rate was highest in Class I, followed by Class II. There was no resolution in Class III (Figure 4). Graft failure was observed in 35% of all cases, with the highest incidence observed in Class III (Figure 5). Subgroup analysis excluding patients with acute rejection (N = 4, three antibody-mediated and one T cell-mediated) showed no change in graft failure rate, most likely due to the small number of patients who had concurrent rejection.

## Discussion

Since discovered in 1971 in the urine of a renal transplant patient [9], polyomavirus infection has gradually increased significantly in the outcome of renal allograft. The latent infection is activated by strong immunosuppressive agents commonly used in modern-day transplant regimens.

The prevalence of PVN in transplant biopsies in our institute is 3.5%, which is similar to a recent study from Australia and New Zealand (3.3%), Europe (4.5%), and the United States (6.6%) [3,10,11]. The decline in PVN incidence over the years in our study is similar to a previous study [10], probably due to more stringent surveillance measures, although the increased incidence in recent years was still reported in the CERTAIN transplant registry [3].

Our study showed a similar distribution of each Banff 2018 PVN histologic class to previous studies [2,6,8,12-14]. Class II was the most prevalent with more than 50% of cases falling into this category. Class I and III were more or less equal in terms of distribution, although more recent studies had a tendency to have more cases with Class I than III [8,15]. This trend likely reflects early disease detection due to standardized screening programs [16]. Class III had the longest time between index biopsies and diagnosis. Significant degree of interstitial fibrosis (ci2, ci3) in this class indicates more chronic and irreversible injury.

The Banff 2018 PVN histologic classification correlated with allograft function, disease resolution, and outcome in our patients. Similar to previous studies [2,12,13], we did not detect an association between demographics, co-morbidities, causes of ESRD, donor type, transplant operation ischemic time, and histologic classification. Class III showed more rapidly elevated serum creatinine and a lesser rate of infection resolution than Class I and II. Although all classes showed no different allograft function at the time of index biopsy, there is a significant elevation of serum creatinine at 12 months post-biopsy. Disease resolution occurred in patients with Class I and II, and no patients with Class III experienced resolution in our study. Patients with Class III also had more graft failure compared to patients with Class I and II. Although graft function and outcome in patients with Class I and II showed no significant difference in the limited follow-up period, Class I showed significantly more resolution compared to Class II. Our study has limitations in terms of a relatively small number of patients, retrospective design, and inclusion of some patients with other concurrent diseases.

## Conclusions

PVN is a common cause of renal allograft failure. Screening for evidence of PVN is now routine in most transplant centers. Histologic diagnosis is still the gold standard for definite PVN diagnosis. The PVN histologic classification in Thai patients provides valuable prognostic information during routine biopsy workup. The classification correlates with allograft function at 12 months and beyond, disease resolution, and allograft loss. Our study supports the validity of the three-tier morphologic PVN classification proposed by the Banff Working Group in 2018.
